# Medicalising normality? Using a simulated dataset to assess the performance of different diagnostic criteria of HIV-associated cognitive impairment

**DOI:** 10.1371/journal.pone.0194760

**Published:** 2018-04-11

**Authors:** Jonathan Underwood, Davide De Francesco, Robert Leech, Caroline A. Sabin, Alan Winston, on behalf of the Pharmacokinetic and Clinical Observations in PeoPle Over fiftY (POPPY) study

**Affiliations:** 1 Division of Infectious Diseases, Imperial College London, London, United Kingdom; 2 Department of Infection & Population Health, University College London, London, United Kingdom; 3 Division of Brain Sciences, Imperial College London, London, United Kingdom; The University of New South Wales, Neuroscience Research Australia, AUSTRALIA

## Abstract

**Objective:**

The reported prevalence of cognitive impairment remains similar to that reported in the pre-antiretroviral therapy era. This may be partially artefactual due to the methods used to diagnose impairment. In this study, we evaluated the diagnostic performance of the HIV-associated neurocognitive disorder (Frascati criteria) and global deficit score (GDS) methods in comparison to a new, multivariate method of diagnosis.

**Methods:**

Using a simulated ‘normative’ dataset informed by real-world cognitive data from the observational Pharmacokinetic and Clinical Observations in PeoPle Over fiftY (POPPY) cohort study, we evaluated the apparent prevalence of cognitive impairment using the Frascati and GDS definitions, as well as a novel multivariate method based on the Mahalanobis distance. We then quantified the diagnostic properties (including positive and negative predictive values and accuracy) of each method, using bootstrapping with 10,000 replicates, with a separate ‘test’ dataset to which a pre-defined proportion of ‘impaired’ individuals had been added.

**Results:**

The simulated normative dataset demonstrated that up to ~26% of a normative control population would be diagnosed with cognitive impairment with the Frascati criteria and ~20% with the GDS. In contrast, the multivariate Mahalanobis distance method identified impairment in ~5%. Using the test dataset, diagnostic accuracy [95% confidence intervals] and positive predictive value (PPV) was best for the multivariate method vs. Frascati and GDS (accuracy: 92.8% [90.3–95.2%] vs. 76.1% [72.1–80.0%] and 80.6% [76.6–84.5%] respectively; PPV: 61.2% [48.3–72.2%] vs. 29.4% [22.2–36.8%] and 33.9% [25.6–42.3%] respectively). Increasing the *a priori* false positive rate for the multivariate Mahalanobis distance method from 5% to 15% resulted in an increase in sensitivity from 77.4% (64.5–89.4%) to 92.2% (83.3–100%) at a cost of specificity from 94.5% (92.8–95.2%) to 85.0% (81.2–88.5%).

**Conclusion:**

Our simulations suggest that the commonly used diagnostic criteria of HIV-associated cognitive impairment label a significant proportion of a normative reference population as cognitively impaired, which will likely lead to a substantial over-estimate of the true proportion in a study population, due to their lower than expected specificity. These findings have important implications for clinical research regarding cognitive health in people living with HIV. More accurate methods of diagnosis should be implemented, with multivariate techniques offering a promising solution.

## Introduction

Cognitive impairment is frequently reported in people living with HIV, apparently affecting up to 50% despite antiretroviral therapy [1,2]. However, the prevalence of cognitive impairment in demographically comparable HIV-uninfected control groups is also reported to be up to 29–36% [3,4]. Such rates of cognitive impairment does not generally tally with clinical experience and symptomatology [5,6]. suggesting that these high rates may be artefactual as a consequence of the approach used to define cognitive impairment.

A frequently used method of defining cognitive impairment in HIV-disease is the HIV-associated neurocognitive disorder (HAND) classification, also known as the ‘Frascati criteria’. This defines an individual as having cognitive impairment if s/he scores one or more standard deviations (SD) below the normative mean in two or more cognitive domains (with the normative means and SDs having generally been obtained from historic population datasets) [7]. Mild impairment is further subdivided into symptomatic and asymptomatic, with those in the two groups classified as having ‘mild neurocognitive disorder’ [MND] and ‘asymptomatic neurocognitive disorder’ [ANI], respectively. HIV-associated dementia, the most severe form of impairment, is defined when an individual scores two or more SDs below the normative mean on tests from at least two cognitive domains with “marked impairment of day-to-day functioning”.

A limitation of the classification of mild impairment (ANI/MND) is the false positive rate: based on this definition and assuming scores follow a normal distribution, approximately 16% (about 1 in 6) of a normative population would be *expected* to score one SD below the normative mean in any of the tests that are performed, even in the absence of any genuine impairment. However, cognitive impairment is rarely diagnosed on the basis of a single test from a single cognitive domain, with assessments of cognitive function usually including multiple tests from multiple different cognitive domains (typically six or seven). Thus, it is highly likely that an individual would be classified as cognitively impaired simply by chance alone. For example, a six domain model of cognitive function testing [3,6] is akin to rolling six dice where each die represents a single fully independent cognitive domain and where a score of ‘one’ is indicative of a value that is >1 SD below the mean. In this case, the expected prevalence of impairment (rolling ‘one’ in at least two of the six dice) is approximately 25%. However, an individual’s performance on different tests of cognition will be correlated, hypothesised to be related to underlying latent factors e.g. general intelligence factor (‘g’) [8]. If cognitive tests are perfectly correlated the probability of cognitive impairment in a normative population is the same as if there was only one test i.e. 16%. However, in a more realistic situation with inter-test correlation coefficients between zero and one, the ‘expected’ prevalence of ‘cognitive impairment’ in a normative population will always exceed 16% [9]. Moreover, this inflated prevalence of ‘cognitive impairment’ would be expected to increase with the number of tests performed if no attempt is made to account for multiple comparisons. This may explain the high reported prevalence of cognitive impairment (30–50%) in HIV-positive cohorts.

An alternative scoring system, known as the global deficit score (GDS), is obtained by converting demographically adjusted test data to deficit scores, averaging them and using a pre-specified threshold (a score ≥0.5) to define as impairment [10]. The purpose of converting demographically adjusted test scores to deficit scores is to attach more weight to impaired performance with less weight placed on scores that are close to, or above, the mean, which could counter-balance impairment if a simple averaging approach across cognitive domains was used. This method aims to be most comparable to clinician rating, the purported ‘gold standard’, and has been shown to have good predictive and discriminatory power in HIV-positive individuals [10]. Furthermore, by averaging over domains it is less affected by multiple comparisons and may be more reliable in terms of test-retest reliability. Considering the ‘expected prevalence’ of impairment in a normative population, if the cognitive tests are perfectly correlated, to have a GDS ≥0.5, the score must be at least one SD below the mean to score a point (no half points are awarded) and therefore the probability of impairment in this scenario is ~0.16 or 16%. If the tests are not correlated, then it is much more complicated as there are many ways to achieve a GDS ≥0.5 (e.g. if testing six domains having a deficit score of three in one domain only or having three domains with a deficit score of only one).

Another method, known as the multivariate normative comparison (MNC), uses a study specific control group as a reference to calculate a multivariate statistic (Hotelling’s *T*^2^) taking into account performance in all cognitive domains and the covariance between tests [11]. As only one statistical test is performed there is no multiple testing. For the diagnosis of HIV-associated cognitive impairment, this method has been shown to potentially optimise the balance between sensitivity and specificity in the absence of a true gold standard [3]. One potential disadvantage of this method is that the relative measure of cognitive impairment provided will depend on the availability and characteristics of the study specific control group–thus, if the study group is compared to a different control group, the relative prevalence of cognitive impairment may change. This contrasts with the Frascati criteria or GDS, which rely on normative means from historical datasets. These are likely to be more comparable between different studies (cognitive batteries and differences in study populations notwithstanding) as the reference group generally remains stable.

At its core, identifying cognitive impairment is akin to outlier detection, namely is this person’s cognitive function below what is expected? For unidimensional data, such as height, the probability of being x number of SDs from the mean can be inferred from the z-distribution. For multidimensional data, such as testing cognitive function with several neuropsychological tests, a multidimensional measure of deviation from the mean is needed–the Mahalanobis distance. Introduced in 1927, to study racial differences in anthropometrics, this statistic can be thought of as a multivariate SD taking into account the covariance between different tests and is related to the Hotelling’s *T*^2^ statistic used in the MNC [12]. Its major limitations are the assumptions of multivariate normality and equal weighting for all variables. Like MNC, this method is not biased by the number of tests performed and is robust to varying correlations between tests. However, in contrast to the MNC method, the Mahalanobis distance can be calculated from the centre of a hypothetical normal population with the same inter-domain correlations as the study population. Therefore, a study-specific control group, although desirable, is not required and prevalences may potentially be compared more fairly between studies. The critical threshold below which participants are defined as having impairment, which for a normally distributed population can be thought of by chance alone, can be fixed *a priori* at an acceptable rate (e.g. the bottom 5^th^ percentile). Therefore, rates above this (i.e. >5%) in a study population would indicate a prevalence that is higher than can be expected by chance alone.

In this paper, we use a simulated dataset informed by real-world cognitive data from the Pharmacokinetic and Clinical Observations in PeoPle Over fiftY (POPPY) study, to assess the performance of several different commonly used approaches to define cognitive impairment, and to compare these to a proposed new multivariate method based on Mahalanobis distance.

## Methods

### Participants

The POPPY study (ClinicalTrials.gov Identifier: NCT01737047, EudraCT Number: 2012-003581-40) is a multi-centre, prospective cohort study primarily investigating the effects of ageing and comorbidities in HIV-positive individuals in the UK and Ireland. For these analyses, participants were prospectively enrolled into the POPPY study at seven sites across the UK. Inclusion and exclusion criteria have been described previously [5,6]. For the purposes of this analysis only HIV-positive and HIV-negative participants aged >50 were included.

All participants provided written informed consent. The study was approved by the UK National Research Ethics Service (NRES; Fulham, London, UK—reference number 12/LO/1409).

### Cognitive function testing

All participants underwent cognitive function testing using a computerised battery (CogState™, CogState Ltd, Melbourne, Australia) covering six cognitive domains including visual learning, psychomotor function, visual attention, executive function, verbal learning and working memory (see Table 1 for details of tests performed and how they map to each cognitive domain) as previously described [5,6]. This has been shown to be a sensitive diagnostic tool for the assessment of HIV-associated CI and allows standardised assessment across sites to be completed in a reasonable amount of time [13,14].

**Table 1:** Cognitive tests administered in the POPPY study by cognitive domain with data transformations.

Cognitive domain	Test administered	Scoring system
**Attention/Working Memory**	One back task	Arcsine root-proportion correct
	Two back task	Arcsine root-proportion correct
**Executive Function**	Groton Maze Learning test	Total errors
	Set shifting task	Total errors
**Processing speed**	Detection task	Log reaction time (ms)
**Visual Attention**	Identification task	Log reaction time (ms)
**Verbal Learning/Memory**	International Shopping list task	Total correct
	International Shopping list task–delayed recall	Total correct
**Visual Learning/Memory**	Continuous paired associate learning test	Total errors
	Groton Maze Learning test–delayed recall	Total errors
	One card learning task	Arcsine root-proportion correct

Raw test scores were log-transformed or arcsine root–transformed where necessary (as recommended by the CogState guidelines for analysis) and converted into demographically-adjusted T-scores (with a mean of 50 and a standard deviation of 10) using the HIV-negative control group as the reference population. This method was used as the CogState norms do not cover the age range of the participants in our study. These adjusted T-scores took into account age, level of education, gender and ethnicity as appropriate. A single T-score was calculated for each of the 6 cognitive domains by averaging individual T-scores within each domain. A global T-score was also obtained by averaging across the six domains. For all T-scores, higher scores indicate better cognitive function. Data integrity and quality checks were applied to ensure that scores were generated from completed and fully-understood tasks for each subject. Individual test scores not meeting integrity and quality checks were excluded.

### Multivariate assessment of cognitive impairment

This method is related to the MNC method described by Huizenga *et al*,[11] however it does not necessarily require a study-specific control group. To estimate the measure, a matrix of correlation coefficients of the study sample’s cognitive data is first calculated. Next, for each subject, the Mahalanobis distance is calculated from a hypothetical control population in which the cognitive data are assumed to follow a multivariate normal distribution with known means and covariance. For T-scores, the normative mean is 50 and SD is 10 for each domain. The population covariance is estimated from the study sample (or control group) by converting the previously calculated correlation matrix into a covariance matrix. The Mahalanobis distance is then calculated for each study participant. As the Mahalanobis distance has magnitude and not direction, it cannot be assumed that larger values correspond to more severe cognitive impairment. Thus, to provide direction, the sign of the difference between the global T-scores (i.e. the means of the domains) of the subject and the population mean are applied to the Mahalanobis distance, so that positive values represent scores in general that are above the mean and negative values represent scores below (i.e. impairment). To determine impairment each subject’s signed Mahalanobis distance is then compared to a critical value.

The critical value can be determined mathematically in two ways, depending on whether the study sample is used to estimate the comparator covariance or if this is determined from an independent reference population (i.e. a control group). In the first instance, the distribution of the squared Mahalanobis distance from the independent multivariate mean approximates the *F* distribution and the following formula can be used:

$$critical\;value=-\sqrt{\frac{F{\;}_{\alpha }{}_{;\;p,n-p}\;\cdot p\left(n-1\right)\left(n+1\right)}{n\left(n-p\right)}}$$

Where:

*n* = the number of subjects.

*p* = the number of domains/tests.

*F* = the critical value from the F distribution with p and n-p degrees of freedom with α = 0.05 (i.e. corresponding to the bottom 5^th^ percentile of a normative population).

If no control group is present, or if the assessment of impairment of members of the control group is to be performed, then each study participant’s Mahalanobis distance will be calculated from the hypothetical control population. To accomplish this, the study observations are used to estimate the covariance for the hypothetical control population. In this case, the squared Mahalanobis distance approximates the β distribution and the following formula can be used:

$$critical\;value=-\sqrt{\frac{{\left(n-1\right)}^{2}}{n}\cdot \beta_{{{}_{{}_{{}_{\alpha }}}}_{;\frac{p}{2},\;\frac{\left(n-p-1\right)}{2}}}}$$

Where:

*n* = the number of subjects

*p* = the number of domains/tests

β = the critical value from the β distribution with parameters $\frac{\text{p}}{2}\text{ and }\frac{\left(\text{n}-\text{p}-1\right)}{2}$ with α = 0.05 (i.e. corresponding to the bottom 5^th^ percentile of a normative population)

**Fig 1:**
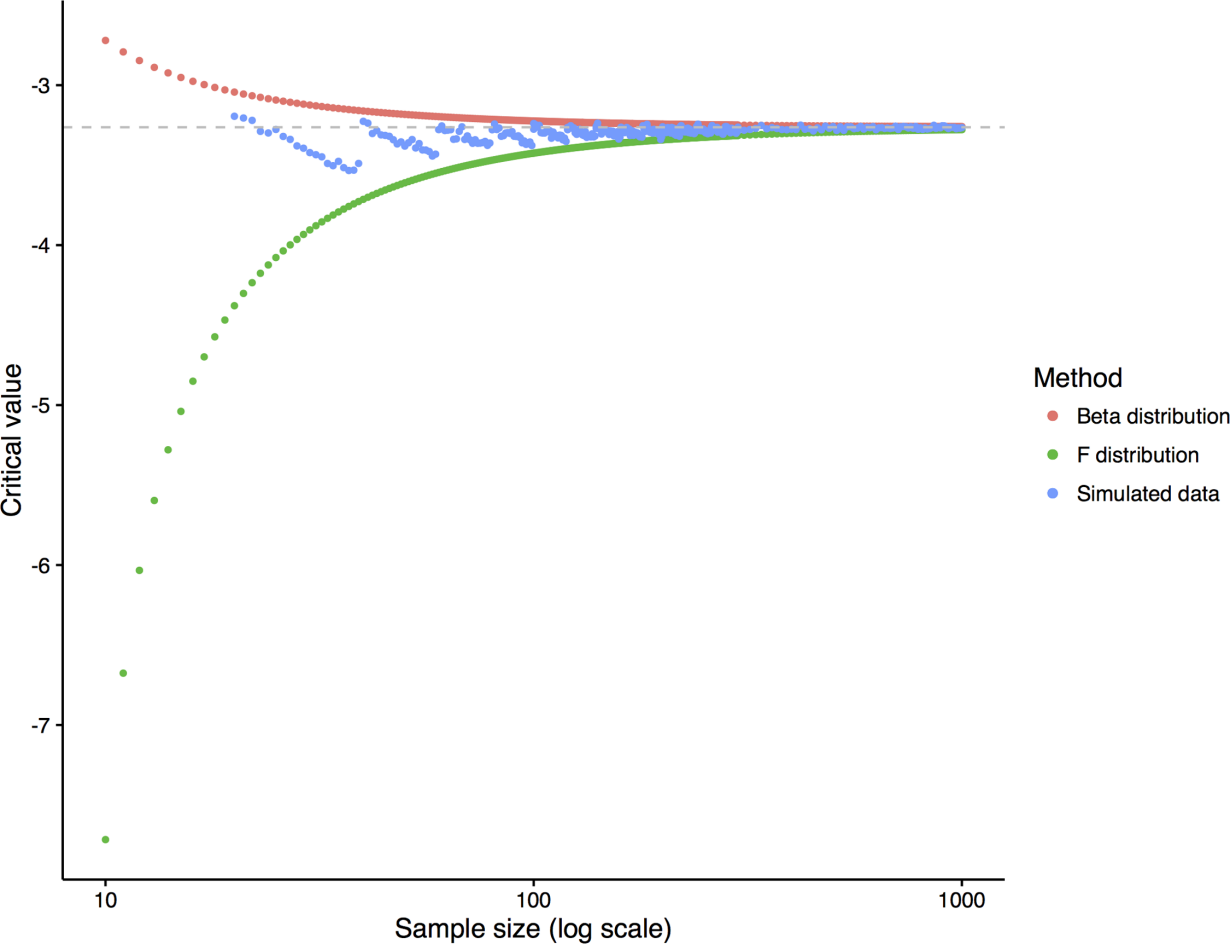
Convergence of the critical value to determine cognitive impairment as the sample size increases. This model assumes a six test/domain model. The critical value from simulated data was the mean of 100 replicates.

These formulae are derived from the equations described by Maesschalck *et al* [12] taking account of the signing process to determine the direction of deviation from the norm and that the base formulae relate to the squared Mahalanobis distance. It should be noted that the first equation using the *F* distribution is very similar to that described for the MNC between two groups described by Huizenga *et al* [11]. Further, they are only appropriate in the context of a multivariate normal distribution (i.e. not significantly skewed/transformed, in which case simulated data and bootstrapping may be more appropriate). Note, where the number of subjects (n) greatly exceeds the number of tests (p) and is large (>200) these methods converge, e.g. for n = 100,000, the critical values are -3.2626 and -3.2628 using the β distribution and *F* distribution (Fig 1).

### Testing the definitions of cognitive impairment

As no gold standard exists for the diagnosis of HIV-associated cognitive impairment, performance of different definitions of cognitive impairment was assessed using simulation informed by real world data. Firstly, the inter-domain correlation coefficients were calculated for both the HIV-positive and HIV-negative groups from the POPPY study. Steiger’s method [15] was used to assess whether the resulting correlation matrices differed between groups. Next, data were simulated for a hypothetical normative ‘control’ population with normally distributed (mean T-score: 50; SD: 10) cognitive scores across six domains using the ‘*mvrnorm*’ command in the MASS [16] package of *R*. These simulated data therefore followed a multivariate normal distribution with inter-domain correlations set to replicate the HIV-positive group from the POPPY study. As a study population invariably involves sampling a subset from the entire population, the sample prevalence of cognitive impairment only approximates the population prevalence when *n* is large. Therefore, a sample (n = 290) was drawn from the simulated normative control population. The Frascati criteria [7], GDS [10] using the threshold of a mean deficit score ≥0.5 to signify impairment and multivariate method outlined above were then applied to calculate the sample prevalence of cognitive impairment for each approach.

Next, an ‘impaired’ population was added to this hypothetical normative control population to create a ‘test dataset’. The ‘impaired’ population were assumed to have a mean T-score of 30 and SD of 10 and thus is comparable to the inclusion of a group of patients with HIV-associated dementia (scores of two SDs or more below the normative mean in 2 or more tests/domains) [7]. The prevalence of ‘impairment’ (i.e. the size of the ‘impaired’ population that was added) was initially set at 10%. Inevitably there is some overlap between those who are labelled as impaired and those on the lower end of the normal distribution. This is likely to be the case in real life, whereby an individual with above average cognitive performance who sustains a brain injury may subsequently perform at an average level.

To assess performance the sensitivity, specificity, predictive value and accuracy were determined by comparing the subjects diagnosed as impaired by each method to the subjects with ‘true’ impairment (true positives). Bootstrapping was performed (10,000 repetitions with replacement) to determine the mean prevalence of cognitive impairment and performance characteristics with 95% confidence intervals. Additionally, separate simulations were performed with different critical values for the Mahalanobis distance method, corresponding to the bottom 10^th^ and 15^th^ percentiles of a normative population. As a final step, the prevalence of true impairment was varied from 0–40% by increasing the size of the ‘impaired’ sample, and the previous steps were repeated.

To provide a clear illustration of the approach, an interactive web-based simulation with adjustable parameters can be found here:

https://jonathan-underwood.shinyapps.io/cognitive_impairment_comparison/

In addition, a web-based tool, where users can upload data and perform standard and multivariate analyses is provided here:

https://jonathan-underwood.shinyapps.io/cognitive_calculator/

Initial collation and analysis of cognitive data was performed using SAS v9.4 (SAS Institute Inc., Cary, NC, USA). All simulations were performed using R v3.2.4 (*R* Foundation for Statistical Computing, Vienna, Austria) with the ‘MASS’ package v7.3–45. The code for these simulations can be found in the supporting information (S1 File). Only P-values (two-sided) <0.05 were considered statistically significant.

## Results

**Fig 2:**
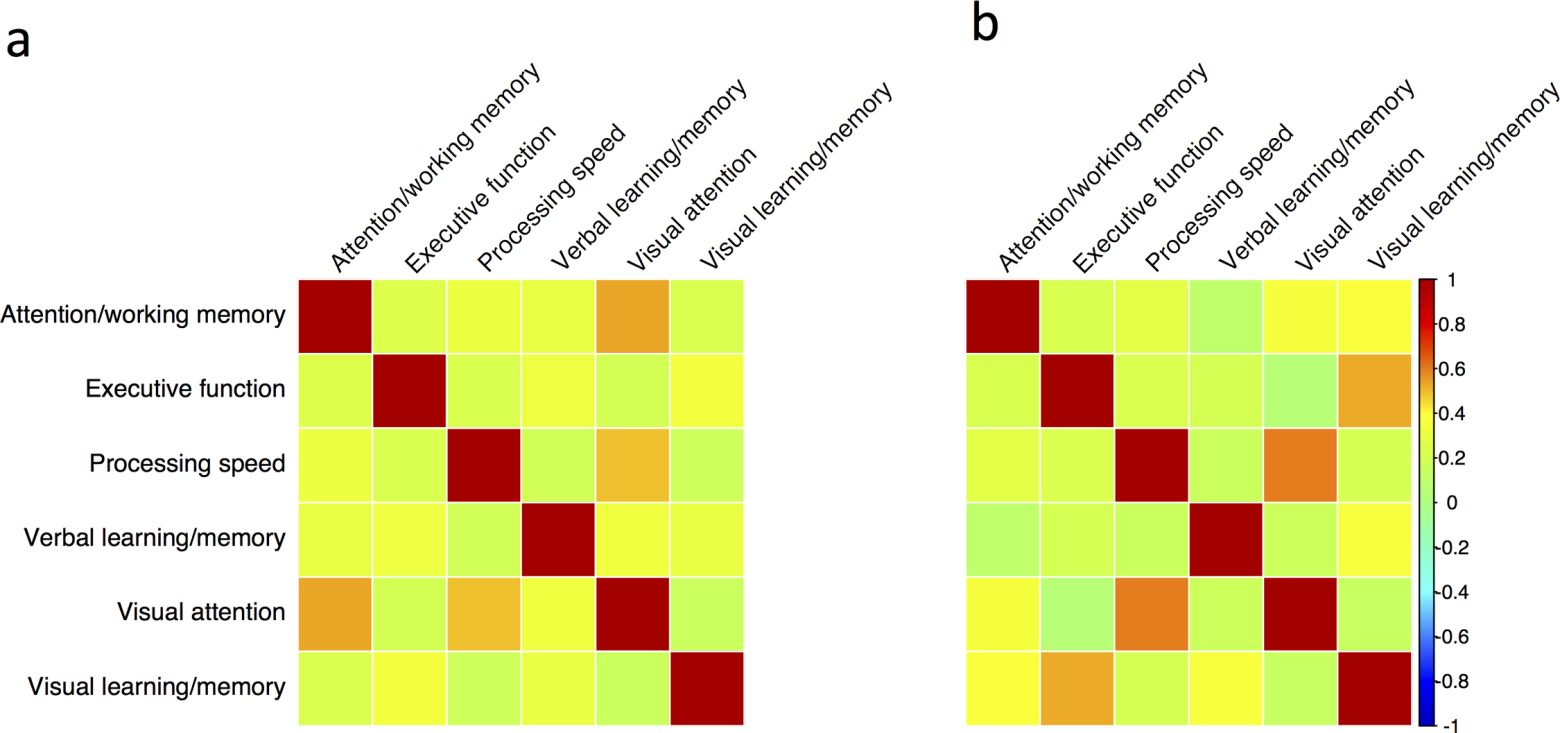
Comparison of the cognitive domain correlation matrices for the HIV-positive and HIV-negative control groups from the POPPY study. Visualisation of the inter-domain correlation matrices for the HIV-negative (panel a) and HIV-positive (panel b) participants of the POPPY study. Colour scale determined by Pearson’s r (scale to the right of the figure).

The inter-domain correlation coefficients for the POPPY HIV-positive group ranged from *r* = 0.05 for processing speed and verbal learning/memory to *r* = 0.60 for executive function and processing speed (Fig 2). The inter-domain correlation matrices did not differ significantly between the HIV-positive and HIV-negative control groups (χ^2^_15_ = 19.5, p = 0.19). The prevalence of cognitive impairment (95% confidence intervals) in the ‘normative’ control population informed by this data was 25.8% (21.7–30.0%) for Frascati; 20.6% (16.9–24.5%) for GDS and 5.0% (3.1–7.2%) for the multivariate Mahalanobis distance method.

**Fig 3:**
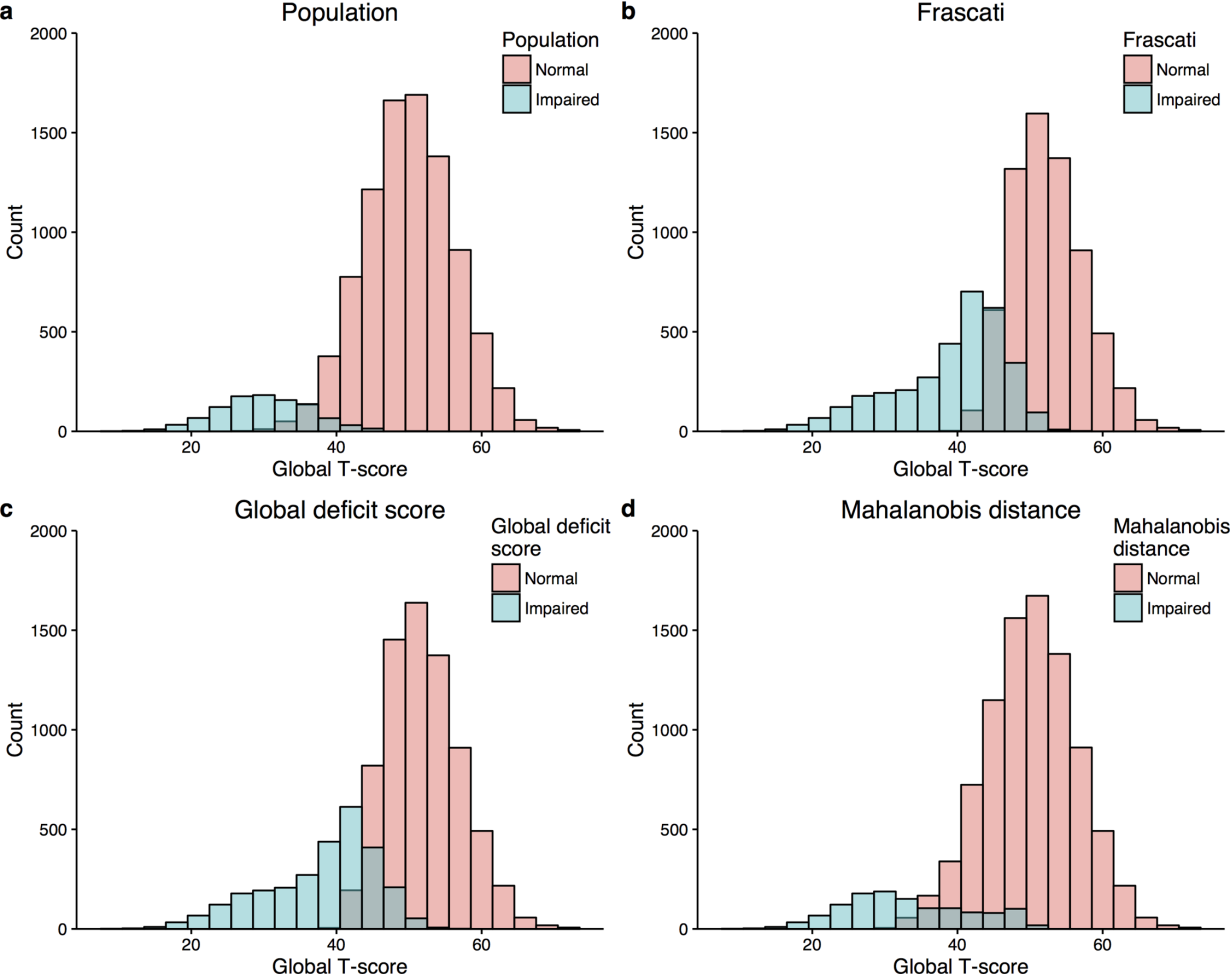
Histograms of a simulated study population with a 10% prevalence of cognitive impairment. Panel a) 90% of the population are ‘normal’ and have a mean (standard deviation) T-score of 50 (10)–red. 10% of the population are impaired and have a mean T-score of 30 (10). Panels b-d: how the population is labelled by method used to define impairment.

When this simulated dataset was enriched with an ‘impaired’ sub-population to create the ‘test’ dataset, comprising 10% of the total (Fig 3A for a graphical illustration), the prevalence of cognitive impairment increased to 33.6% (29.3–38.3%) using the Frascati criteria (Fig 3B); 28.5% (24.8–32.8%) for GDS (Fig 3C); and 12.1% (9.0–15.1%) for the multivariate Mahalanobis distance method (Fig 3D and Table 2 for performance diagnostics). Accuracy was best for the Mahalanobis distance method (92.8% [90.3–95.2%] vs. 76.1% [72.1–80.0%] for Frascati and 80.6% [76.6–84.5%] for the GDS). Accuracy was non-significantly improved using a definition of impairment whereby participants had to be identified by all three definitions over the Mahalanobis distance method on its own (93.6% [91.0–95.9]).

**Table 2:** Performance of three definitions of cognitive impairment in a simulated population with a 10% prevalence of cognitive impairment.

Criteria	Sensitivity (95% CI)	Specificity (95% CI)	PPV (95% CI)	NPV (95% CI)	Accuracy (95% CI)
**Frascati**	98.7% (94.7–100%)	73.5% (69.0–78.0%)	29.4% (22.2–36.8%)	99.8% (99.1–100%)	76.1% (72.1–80.0%)
**Global deficit score**	98.2% (93.3–100%)	78.7% (74.4–82.7%)	33.9% (25.6–42.3%)	99.7% (99.0–100%)	80.6% (76.6–84.5%)
**Mahalanobis distance**	77.4% (64.5–89.4%)	94.5% (92.8–95.2%)	61.2% (48.3–74.2)	97.4% (95.7–98.8%)	92.8% (90.3–95.2%)
**Combination of all three**	77.4% (64.5–89.5%)	95.4% (93.2–97.3%)	65.2% (51.6–78.4%)	97.4% (95.7–98.8%)	93.6% (91.0–95.9%)

Reducing the critical value used to define impairment using the Mahalanobis distance method resulted in higher sensitivity at a cost of specificity (Table 3). However, accuracy was still superior to the Frascati and GDS methods of defining impairment with a threshold set to the bottom 15^th^ percentile of a normative population.

**Table 3:** Performance of five definitions of cognitive impairment in a simulated population with a 10% prevalence of cognitive impairment.

Criteria	Sensitivity (95% CI)	Specificity (95% CI)	PPV (95% CI)	NPV (95% CI)	Accuracy (95% CI)
**Frascati**	98.7% (94.7–100%)	73.5% (69.0–78.0%)	29.4% (22.2–36.8%)	99.8% (99.1–100%)	76.1% (72.1–80.0%)
**Global deficit score**	98.2% (93.3–100%)	78.7% (74.4–82.7%)	33.9% (25.6–42.3%)	99.7% (99.0–100%)	80.6% (76.6–84.5%)
**Mahalanobis distance (alpha 5%)**	77.4% (64.5–89.4%)	94.5% (92.8–95.2%)	61.2% (48.3–74.2)	97.4% (95.7–98.8%)	92.8% (90.3–95.2%)
**Mahalanobis distance (alpha 10%)**	87.8% (77.1–96.7%)	90.0% (86.9–93.0%)	49.5% (38.0–61.1%)	98.5% (97.1–99.6%)	89.8% (87.0–92.8%)
**Mahalanobis distance (alpha 15%)**	92.2% (83.3–100%)	85.0% (81.2–88.5%)	40.6% (30.9–50.7%)	99.0% (97.9–100%)	85.7% (82.4–89.0%)

**Fig 4:**
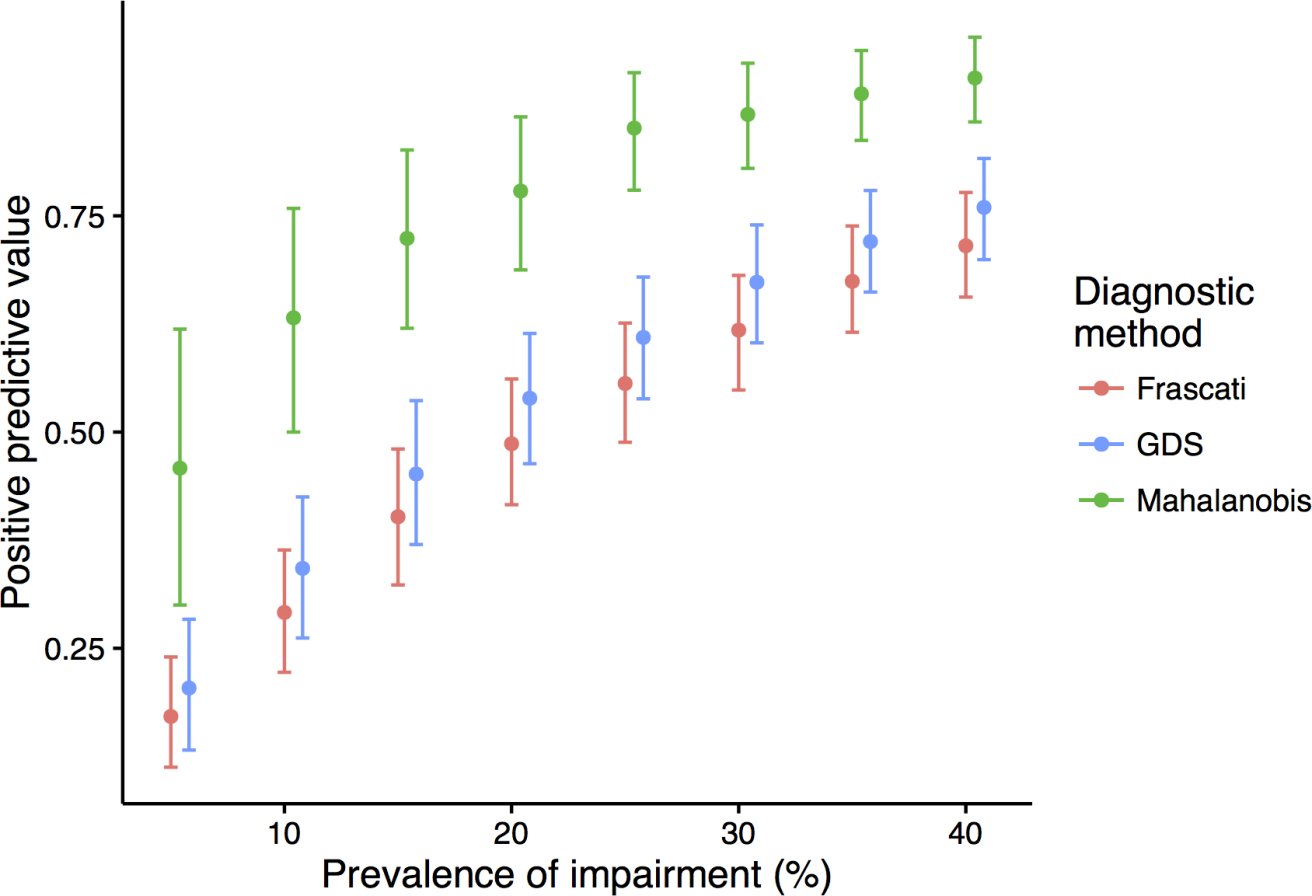
Positive predictive value of each diagnostic method by prevalence of impairment in the simulated study population.

When the prevalence of impairment was 5% in the test dataset, the positive predictive value of true cognitive impairment was below 25% for the Frascati and GDS compared with nearly 50% for the Mahalanobis distance method (Fig 4). As expected, as the prevalence of impairment increased in the test dataset the positive predictive value increased for all three methods, but was significantly higher for the Mahalanobis distance method across the range of prevalences tested (5–40%).

## Discussion

Here, we have shown the utility of using a simulated dataset to test the performance characteristics of two commonly used methods of defining HIV-associated cognitive impairment. Using real-world cognitive data to inform our multivariate model, the Frascati and GDS methods would classify over 20% of a normative control population as impaired. Using the multivariate approach described here, this can be limited to any predetermined level and has demonstrably superior accuracy and positive predictive value.

Standard deviations are useful when dealing with one dimensional, normally distributed data. However, with correlated multivariate data, they are less useful and arguably inappropriate. This is particularly true if there is no stipulated maximum number of tests that a given criterion is applied to. Therefore, it is not unsurprising, given the methodology of both the Frascati criteria and GDS, that such a large proportion of a normative control population are classified as impaired by chance alone. Comparable prevalences of cognitive impairment have been reported in HIV-negative control groups (36% [3] and 29% [4]) of two recent studies. Similarly, McDonnel *et al* [4] reported impairment in 27% of their HIV-negative control group using the GDS. As we have demonstrated, these estimates are similar to what would be expected by chance. Our findings potentially have relevance in other fields (and could be easily applied to any non-cognitive multivariate data). For example, ageing-associated cognitive decline (AACD) uses a similar definition as the Frascati criteria to determine impairment. A study [17] in a healthy elderly population identified AACD in 26.6%—a figure that would be expected by chance as demonstrated here.

It should be noted that comparing neuropsychological test scores to a numerical threshold is not the sole arbiter of whether a patient is labelled as cognitively impaired or not. It is imperative that potentially confounding comorbidities and functional status are also taken into account. It is also essential to understand the properties of any statistical procedure that is used to determine abnormal performance. As demonstrated here, the Frascati definition of abnormal testing will label about a quarter of a healthy reference population as cognitively impaired. This is in agreement with previous work by Taylor & Heaton[18] who found that to maintain specificity with a six domain battery, impairment of >1SD in three or more domains would be necessary. Given that asymptomatic impairment has by definition no functional impact, a quarter of the normative reference population will therefore meet the criteria for ANI. Therefore, even if HIV is not associated with any degree of brain injury one would expect ~25% to be labelled as having ANI (and ~2% to meet the neuropsychological testing criteria for HIV-associated dementia) by chance variation alone. These high estimates of HIV-associated cognitive impairment are problematic for several reasons. Firstly, without a control group for comparison, the prevalence of impairment may be substantially over interpreted, with assumptions made about a causal impact of HIV on cognition. Secondly, such misclassification may hamper studies of the pathophysiology of disease, as many individuals may have been labelled as impaired by chance alone, leading to a reduced ability and power to detect associations with other measures of interest (e.g. soluble biomarkers associated with specific pathogenic processes). Thirdly, the use of these methods as either inclusion criteria or as outcome measure, may reduce the ability of a clinical trial to demonstrate a beneficial effect of an intervention. Finally, if such research methods are used in clinical practice to identify patients with cognitive impairment, it may lead to unnecessary anxiety in those who are falsely labelled as having impairment and may prompt unnecessary further investigations or treatment. Hence there is a need for a robust method of defining cognitive impairment which will not over-inflate the false positive rate.

For the purposes of determining the pathophysiology of HIV-associated cognitive impairment, it may be advantageous to use continuous measures of cognitive function. Dichotomising data into two groups for statistical analysis has many drawbacks [19]. Firstly, given the distribution of most cognitive data, the assumption there are two clear groups is probably not valid. Furthermore, the difference between those with and without impairment at the margin, is greatly exaggerated. Secondly, as up to a third of information is lost [19], statistical power is reduced which may lead to false conclusions being drawn. With these shortcomings, it is worth considering why a standardised definition of cognitive impairment is required. Scientific curiosity aside, the purpose of a definition is generally to aid the treatment of disease. At some point a yes/no or impaired/not impaired decision must be made and thus dichotomised cognitive function data is desirable. The motivation for updating the HIV-associated neurocognitive disorders classification, to create the so-called Frascati criteria, was to improve standardisation by introducing clear definitions of what was considered abnormal and to include a category of asymptomatic impairment [7]. Prior to this update in 2007, the study of HIV-associated cognitive impairment focused on severe dementia, the prevalence of which declined dramatically with the advent of antiretroviral therapy. At the time the HAND criteria were proposed they took into consideration the changing phenotype of cognitive disorders in HIV-disease. This category of mild impairment, without deficit in performance in everyday activities, was thought to be a possible precursor to more severe impairment. Consequently, identification of patients in this group was considered important as subsequent intervention may prevent further decline. However, the evidence that these mild forms of cognitive impairment progress is mixed and the data that do suggest an increased risk of functional impairment longitudinally were in populations with inadequate suppression of HIV-replication [20–23]. Nearly a decade after its introduction, the phenotype of HIV-associated cognitive disorders is again changing as antiretroviral therapy has improved and patients are ageing. Our simulation data suggests that this definition, as it stands, is insufficiently stringent, with many patients being labelled as impaired by chance. The GDS method represents a slight improvement, but still labels approximately 20% of a normative control population as impaired. A potentially better, albeit slightly more complicated, method using the multivariate Mahalanobis distance allows the false positive rate to be controlled at any desired level. As can be seen in table three, increasing the false positive rate (alpha) from 5% to 15% and therefore decreasing the specificity from 95% to 85%, results in an increase in sensitivity at a cost of accuracy. Choosing the optimal threshold requires a compromise between false positive and false negative tests, but in general the specificity should be high, sensitivity and specificity should be balanced and accuracy should be high as possible. [18] The optimal false positive rate for the study of HIV-associated cognitive impairment is unknown but could be tested in future studies using neuroimaging and CSF biomarkers of brain injury. One potential limitation of this method is that the covariance between cognitive domains is estimated from the patient group, which may differ from a control group. However, whilst having a control group is preferable, the correlation matrices between patient and control groups did not differ significantly in the POPPY study, justifying this approach. Another potential limitation of this model is that it is based on inter-domain correlations obtained from study data where participants were tested using the CogState™ battery, which has not been externally or clinically validated for its embedded cognitive constructs. Further study of this novel definition of cognitive impairment in other cohorts, where different neuropsychological test batteries have been used are warranted. Furthermore, the associations between neuroimaging and CSF biomarkers and this definition of cognitive impairment are needed to assess whether tighter associations between pathogenic mechanisms are observed more consistently than with previous methods. Web-based tools are provided to illustrate the approach and allow it to be easily applied to other datasets:

https://jonathan-underwood.shinyapps.io/cognitive_impairment_comparison/ and https://jonathan-underwood.shinyapps.io/cognitive_calculator/.

### Conclusion

The commonly used diagnostic criteria for HIV-associated cognitive impairment do not account for the correlation structure known to exist across cognitive tests. Our simulations show that they label a significant proportion of a normative reference population as cognitively impaired, which will likely lead to a substantial over-estimate of the true proportion in a study population, due to their lower than expected specificity. These findings have implications for future research. More statistically appropriate methods of diagnosis should be considered, with multivariate techniques offering a promising solution.

## Supporting information

S1 File R code for simulations. (DOCX)
