# Supplementary material for: Medicalising normality? Using a simulated dataset to assess the performance of different diagnostic criteria of HIV-associated cognitive impairment
Source: PLoS One. 2018 Apr 11;13(4):e0194760. doi: 10.1371/journal.pone.0194760 (PMC5894993; doi:10.1371/journal.pone.0194760)
Supplement: S1 File — (DOCX) [file pone.0194760.s001.docx]

simpop_with_impaired.R

jonathanunderwood

Tue Apr 4 22:19:22 2017

# A comparison of two methods of diagnosing HIV-associated cognitive impairment
# with a new, multivariate approach inspired by the work of Huizenga et al
#
# Jonathan Underwood (v1.0 December 2016)
#
# For research purposes only
# If this is used in your research please cite our paper - thanks

require(MASS)
require(psych)
sigma <-matrix(c(1,0.210754443741029,0.145349600511535,0.528406352623827,0.367212674407502,0.377429548420058,
 0.210754443741029,1,0.601043770264608,0.233912234063957,0.156447757547805,0.27289232693919,
 0.145349600511535,0.601043770264608,1,0.0580374512388783,0.166080151414515,0.357473226464959,
 0.528406352623827,0.233912234063957,0.0580374512388783,1,0.210596654179787,0.222291349676154,
 0.367212674407502,0.156447757547805,0.166080151414515,0.210596654179787,1,0.100172102842648,
 0.377429548420058,0.27289232693919,0.357473226464959,0.222291349676154,0.100172102842648,1), nrow = 6)
syn_n=100000
mean=50
mean2=30
sd=10
sd2=10

sample_n <- 290 # study n
reps <- 10000 #replicates
covar <- sigma * sd^2
means <- rep(50,6)
alpha <- 0.1 # one sided 0.05
p <- nrow(sigma)
critical <- -3.249432
# -sqrt((qf(p = 1- alpha, df1 = p, df2 = sample_n-p) * (sample_n+1) * p*(sample_n-1))/((sample_n-p)* sample_n))
# -sqrt(qbeta(1-alpha,p/2, (sample_n-p-1)/2 ) * ((sample_n-1)^2)/sample_n)

results_HAND <- NULL
results_GDS <-NULL
results_mahal <- NULL
results_HAND.kappa <- NULL
results_GDS.kappa <- NULL
results_mahal.kappa <- NULL
results_HAND.sens <- NULL
results_HAND.spec <- NULL
results_HAND.acc <- NULL
results_HAND.ppv <- NULL
results_HAND.npv <- NULL
results_GDS.sens <- NULL
results_GDS.spec <- NULL
results_GDS.acc <- NULL
results_GDS.ppv <- NULL
results_GDS.npv <- NULL
results_mahal.sens <- NULL
results_mahal.spec <- NULL
results_mahal.acc <- NULL
results_mahal.ppv <- NULL
results_mahal.npv <- NULL
results_all <- NULL
results_all.sens <- NULL
results_all.spec <- NULL
results_all.acc <- NULL
results_all.ppv <- NULL
results_all.npv <- NULL

for(a in seq(5,40,5)){
 print(paste("Now simulating ", reps, " repititons of ", sample_n, " patients with a population prevalence of impairment of ", a, "%", sep = ""))
 syn_n*(a/100) -> n2
 syn_n-n2 ->n
 data.frame((mvrnorm(n, mu = rep(0,nrow(sigma)), Sigma = sigma,empirical = TRUE) * sd) +mean) -> synthetic_pop2
 rep(1,n) -> synthetic_pop2$label

 data.frame((mvrnorm(n2, mu = rep(0,nrow(sigma)), Sigma = sigma,empirical = TRUE) * sd2) +mean2) ->synthetic_pop2.imp
 rep(2,n2) -> synthetic_pop2.imp$label

 rbind(synthetic_pop2, synthetic_pop2.imp) ->synthetic_pop2

 for(i in 1:reps) {
 synthetic_pop2[sample(nrow(synthetic_pop2),size = sample_n, replace = F),] -> test
 apply(test[1:6],1,function(x) sum(x<40)>1) -> HAND # generates frascati
 apply(test[1:6],1, function(i) sign(mean(i)-mean)*sqrt(mahalanobis(i,center = means, cov = covar))) -> mahal_dist # generates signed mahal distance

 apply(test[1:6],2,function(x)
 ifelse(x>19,
 ifelse(x>24,
 ifelse(x>29,
 ifelse(x>34,
 ifelse(x>40,0,1),2),3),4),5)
 ) -> test2
 apply(test2,1,function(x) mean(x)>=0.5) ->GDS # generates global deficit score


 rbind(results_HAND, sum(HAND)/sample_n * 100) -> results_HAND
 rbind(results_GDS, sum(GDS)/sample_n * 100) -> results_GDS
 rbind(results_mahal, sum(mahal_dist<critical)/sample_n * 100) -> results_mahal
 rbind(results_all, sum(HAND==1 & GDS ==1 & mahal_dist<critical)/sample_n * 100) -> results_all
 rbind(results_HAND.kappa, cohen.kappa(table( as.matrix(HAND)==1, test$label==2))$kappa) -> results_HAND.kappa
 rbind(results_GDS.kappa, cohen.kappa(table( as.matrix(GDS)==1, test$label==2))$kappa) -> results_GDS.kappa
 rbind(results_mahal.kappa, cohen.kappa(table( as.matrix(mahal_dist<critical)==1, test$label==2))$kappa) -> results_mahal.kappa

 rbind(results_HAND.sens, sum((HAND)==1 & test$label==2)/sum(test$label==2)) ->results_HAND.sens
 rbind(results_HAND.spec, sum((HAND)==0 & test$label==1)/sum(test$label==1)) ->results_HAND.spec
 rbind(results_HAND.acc, (sum(HAND==1 & test$label==2) + sum(HAND==0 & test$label==1))/sample_n) -> results_HAND.acc
 rbind(results_HAND.ppv, sum(HAND==1 & test$label==2)/sum(HAND==1)) -> results_HAND.ppv
 rbind(results_HAND.npv, sum(HAND==0 & test$label==1)/sum(HAND==0)) -> results_HAND.npv

 rbind(results_GDS.sens, sum((GDS)==1 & test$label==2)/sum(test$label==2)) ->results_GDS.sens
 rbind(results_GDS.spec, sum((GDS)==0 & test$label==1)/sum(test$label==1)) ->results_GDS.spec
 rbind(results_GDS.acc, (sum(GDS==1 & test$label==2) + sum(GDS==0 & test$label==1))/sample_n) -> results_GDS.acc
 rbind(results_GDS.ppv, sum(GDS==1 & test$label==2)/sum(GDS==1)) -> results_GDS.ppv
 rbind(results_GDS.npv, sum(GDS==0 & test$label==1)/sum(GDS==0)) -> results_GDS.npv

 rbind(results_mahal.sens, sum(mahal_dist<critical & test$label==2)/sum(test$label==2)) ->results_mahal.sens
 rbind(results_mahal.spec, sum(mahal_dist>critical & test$label==1)/sum(test$label==1)) ->results_mahal.spec
 rbind(results_mahal.acc, (sum(mahal_dist<critical & test$label==2) + sum(mahal_dist>critical & test$label==1))/sample_n) -> results_mahal.acc
 rbind(results_mahal.ppv, sum(mahal_dist<critical & test$label==2)/sum(mahal_dist<critical)) -> results_mahal.ppv
 rbind(results_mahal.npv, sum(mahal_dist>critical & test$label==1)/sum(mahal_dist>critical)) -> results_mahal.npv

 rbind(results_all.sens, sum((HAND==1 & GDS ==1 & mahal_dist<critical) & test$label==2)/sum(test$label==2)) ->results_all.sens
 rbind(results_all.spec, sum((HAND==0 | GDS ==0 | mahal_dist>critical) & test$label==1)/sum(test$label==1)) ->results_all.spec
 rbind(results_all.acc, (sum((HAND==1 & GDS ==1 & mahal_dist<critical) & test$label==2) + sum((HAND==0 | GDS ==0 | mahal_dist>critical) & test$label==1))/sample_n) -> results_all.acc
 rbind(results_all.ppv, sum((HAND==1 & GDS ==1 & mahal_dist<critical) & test$label==2)/sum((HAND==1 & GDS ==1 & mahal_dist<critical))) -> results_all.ppv
 rbind(results_all.npv, sum((HAND==0 | GDS ==0 | mahal_dist>critical) & test$label==1)/sum((HAND==0 | GDS ==0 | mahal_dist>critical))) -> results_all.npv
 }
}


for(i in 0:7){
 i*reps ->i1
 (i+1)*reps ->i2
 print(paste("Population prevalence of impairment of ", (i+1)*5, "%", sep = ""))
 print(apply(data.frame(results_HAND[i1:i2],results_HAND.sens[i1:i2],results_HAND.spec[i1:i2], results_HAND.acc[i1:i2], results_HAND.ppv[i1:i2], results_HAND.npv[i1:i2]), 2, function(x) {
 (c(mean(x), sort(x)[.05*reps], sort(x)[.95*reps]))
 }))

 print(apply(data.frame(results_GDS[i1:i2],results_GDS.sens[i1:i2],results_GDS.spec[i1:i2], results_GDS.acc[i1:i2], results_GDS.ppv[i1:i2], results_GDS.npv[i1:i2]), 2, function(x) {
 (c(mean(x), sort(x)[.05*reps], sort(x)[.95*reps]))
 }))

 print(apply(data.frame(results_mahal[i1:i2],results_mahal.sens[i1:i2],results_mahal.spec[i1:i2], results_mahal.acc[i1:i2], results_mahal.ppv[i1:i2], results_mahal.npv[i1:i2]), 2, function(x) {
 (c(mean(x), sort(x)[.05*reps], sort(x)[.95*reps]))
 }))

 print(apply(data.frame(results_all[i1:i2],results_all.sens[i1:i2],results_all.spec[i1:i2], results_all.acc[i1:i2], results_all.ppv[i1:i2], results_all.npv[i1:i2]), 2, function(x) {
 (c(mean(x), sort(x)[.05*reps], sort(x)[.95*reps]))
 }))
}
